# 加速康复外科——人文or技术?

**DOI:** 10.3779/j.issn.1009-3419.2018.03.08

**Published:** 2018-03-20

**Authors:** 国卫 车

**Affiliations:** 610041 成都，四川大学华西医院胸外科 Department of Toracic Surgery, West China Hospital, Sichuan University, Chengdu 610041, China

**Keywords:** 加速康复外科, 人文, 技术, Enhanced recovery afer surgery, Humanity, Technology

## Abstract

外科技术进步和器械的更新必然促进手术方式的变化，技术的发展导致外科观念更新。加速康复外科（enhanced recovery afer surgery, ERS）的理念使外科的内涵从“治疗疾病”转变为“治病救人”，外延也从“单纯手术”变为“促进康复”。但加速康复外科的理念来源于外科技术进步，但又高于外科技术，体现在更加重视“人”，而将安全和康复置于具体外科治疗之上。主要体现有：一是ERS重视术前多学科评估（以病人为中心），选择合适的（个性化）治疗方案，降低并发症和死亡率；二是ERS重视术前准备（以问题为导向），高危因素的多科协作预防，减少创伤并预防并发症，节约医疗成本；三是ERS强调优化围手术期流程，改变（医务工作者）工作习惯与模式。总之，将ERS理念应用于围手术期从管理到治疗的各个环节，每个环节争取做到“减少应激和创伤”，完美体现“no pain and no risk”的理念。

## Abstract (en)

Te development of surgical techniques and the innovation of surgical instruments promote the changes in surgical methods, which in turn, leads to the reforms of surgical idea. Te concept of enhanced recovery afer surgery (ERS) results in the surgery connotation switching from "to treat an illness" to "to save patients". ERS also causes the change of surgery denotation from "only operation" to "enhance recovery". Te concept of ERS is derived from but not only restricted to the development of surgical techniques. Also, ERS pays more atention to patients than disease and thinks more about patient safety and recovery than surgical technique. Specifcally, ERS has three advantages: frst, ERS ataches importance to multidisciplinary assessment (patient-oriented) and select appropriate (personalized) treatment and tries to decrease the rate of complication and mortality. Second, ERS pays atention to preoperative preparation and high-risk prevention performed by multi-disciplinary collaboration in order to prevent complication and save cost for patients. Tird, ERS always focus on optimizing the process of perioperative period and trying to change the working habits of surgeons and the old cooperation mode among doctors, nurses and rehabilitation physicians. To sum up, the goal is to apply the concept of ERS to all the links of perioperative period and try hard to decrease patients' stress and pain, which fnally helps doctors fulfll the ultimate aims of "no pain and no risk".

微创外科技术和多学科协作极大推动了加速康复外科(enhanced recovery after surgery, ERAS)的临床应用。ERAS理念不但丰富了外科学的内涵，且改善了患者的就医体验。但临床推广应用过程中存在以下事实与倾向^[1]^：一是微创外科技术普及应用的程度远远大于ERAS的理念；二是ERAS的专家共识难以在临床完全实现，很多单位仍难以开展应用；三是部分外科医生过分重视技术在ERAS应用中的作用；如何才能更好的在临床工作中推动ERAS实施呢？首先，正确认识ERAS"no pain and no risk"理念的内涵与外延：内涵是"问题导向，病人为中心"，外延是"技术进步，学科协作"^[2]^。其次是临床工作中医护康的团结协作，优化临床流程和解决问题，从心理到身体减少或消除患者的痛苦^[3]^。ERAS的普及应用将充分体现以人为本、病人的最大利益主导一切外科活动。ERAS关键与核心问题是人文关怀，技术进步只是解决问题的一个手段。总之，ERAS更多体现的是"人文关怀"，而非单纯的技术进步。

## 1 多学科协作使ERS方案"个体化"和"人性化"

ERAS的初心是使患者从心理和身体上的完全康复，外科治疗只是康复的手段而不是目的。需要我们医务工作者从患者"康复角度"而不是从"祛病角度"制订治疗方案。事实上需要外科手术治疗的多数疾病，均需要多学科协作，每个专业都既需要从患者疾病和整体治疗过程全面制订处理方案，避免顾此失彼。而要做到这些，需要所有专科从治疗开始就对患者进行整体考虑，并将围手术期各个阶段治疗方案细化并付诸实施。"个体化"的治疗需要需要因人而已，"人性化"需要预防、治疗与康复并重。如肺癌患者的外科治疗，术前多学科评估可以将需要外科手术治疗的患者大致分为三类："正常"、"高危"和"症状"人群^[4]^。"正常"肺癌患者是指即年龄小于60岁，且体检发现的小结节，无明显伴随疾病的患者；其ER AS方案是以微创技术的合理应用为核心，优化手术相关流程为步骤，以缩短平均住院日或日间手术能否运用作为评价标准；可优化的流程主要有：麻醉是否需要气管插管(根据医院情况而定)；管道(引流管和尿管等)及镇痛的合理应用，可以使患者早期下床活动并促进胃肠功能快速恢复^[5]^。"症状"肺癌患者是指术前患者具有临床症状或出院后发生的与手术相关的常见症状(如咳嗽、气短和疼痛等)^[5]^，以控制症状为主的术前治疗措施或基于术后症状而对手术技术或过程改进或优化为关键(预防)，以改善患者生活质量为核心。"高危因素"患者主要是指因患者自身或医疗相关因素导致围手术期并发症或死亡率增加^[5]^。自身因素主要与年龄、生活习惯(如吸烟等)和合并疾病(如慢性阻塞性肺疾病、高血压等)；医疗因素主要与手术过程相关，如麻醉时间过长(包括手术时间长)，手术创伤(术中肺挫裂伤，失血或输液过多等)，而外科因素常常因各种原因被忽视^[5]^。肺癌患者合并高危因素患者的ERS方案的核心是围手术期肺康复训练，同时医疗因素是优化流程和加强管理^[6]^。

多学科以病人为中心的"虚拟团队"建设也是将ERAS方案"个体化"的过程，外科不但是切除肿块，更加看重的是患者躯体与心理的康复，充分体现了"人性化"关怀。ERAS方案更多的是技术的整合与优化，只是运用了适宜于个体患者的治疗方案。

## 2 ERS理念使预防、康复和治疗并重

ERAS的核心是减少创伤和应激，体现治疗过程的安全性。客观和准确的评估才能保证"个体化或精准化" ERAS方案的顺利、有效的执行^[7]^。优化流程主要是治疗手段尽量不扰动机体的生理机能，预防因操作导致的不必要损伤；如麻醉是否需要气管插管，若行气管插管则必然对气道造成损伤，术中清扫肿大淋巴结是否可以考虑不切断周围神经(如迷走神经)和重要滋养血管(食管动脉)等。再如尿管的应用，主要是预防尿潴留，若ERAS方案中评估患者手术时间短，减少液体输入，则就可不必安置；若患者高龄且前列腺中度以上增生，术前应用盐酸坦索罗辛缓释胶囊(哈乐等)，是否就可以避免留置尿管^[8, 9]^。胸腔镜肺癌患者术后疼痛主要部位为胸腔引流管口，疼痛性质为胀疼，考虑主要原因为引流管粗需缝线固定或引流管拔出后的预置线结扎导致，缓解或降低疼痛发生主要是改进胸腔引流的相关措施，而不是过分依赖镇痛药^[10-12]^。这些都是从预防的角度采取的ERAS方案，减少不必要创伤，可以提高就医舒适度和满意度。

多学科协作主要是预防术后并发症及影响生活质量的症状发生，如肺癌患者术前合并自身疾病(如高血压或中重度慢性阻塞性肺疾病等)或症状的治疗不但有助于降低术后并发症，也有助于提高住院舒适度和术后生活质量^[13-15]^。肺癌患者出院后导致咳嗽的危险因素为术前有咳嗽症状和麻醉时间长^[16-18]^。通过围手术期的肺康复训练，可以有效降低肺癌患者术后咳嗽的严重程度^[19]^。疲劳与气短也是影响肺癌患者术后生活质量改善的主要因素，术后发生气短的肺癌患者与肿瘤分期晚、手术范围大，及肺功能相对差有关，围手术期相应的肺功能训练，可以有效降低术后气短的发生程度^[20]^。多学科团队协作可以有效将最适合于患者的方案应用于围手期治疗和各个环节，充分体现预防与治疗并重的ERS理念。

## 3 ERS临床实践的思维模式和工作习惯更加彰显人文性

目前出版的各种ERAS专家共识及指南均将取得患者配合是ERAS成功的关键。ERAS的术前宣教更加着重人文关怀，主要表现在：①护士从"教条式"宣讲到"教育式沟通"的转变；②患者从"从被动式接受"到"主动参与"的转变；③医生从"事后性处理"到"事前性预防"式转变；④从"单一外科"到"多学科"沟通；这些变化体现的不是高大上的技术进步，是以"问题为中心"医生、护士和患者共同参与的诊疗过程，是以"病人为中心"的平等交流与协作的医疗体验。在和患者沟通ERAS方案时，也改变着团队的思维模式和工作习惯。

术前宣教主要是由护士来完成，多数情况下偏重于事务性及流程性介绍，很少涉及医疗过程出现问题的宣讲。主要原因是一方面是护士自身很少主动参与治疗过程，导致护士整个治疗过程出现的问题不熟悉，体会不深刻而讲不清楚；另外，患者自身也缺乏对护士工作的信任；而导致"不愿听，我也不愿讲"的恶性循环^[21]^。如何才能建立信任并有效的进行宣教呢？首先，护士要避免进行"空对空"和"教条式"的宣讲，ERAS方案实施后，护士通过参与治疗方案的实施，使术前宣教针对性强，事务性宣讲减少，而偏重于科室患者的共性问题，如医疗过程中各级医生的职责及发生问题时处理流程等，建立起相互信任。尤其是许多医院都会在护士术前宣教中加入肺康复训练的相关措施，患者从"被动式接受"到"主动参与"方案的实施如：正确的咳嗽方法，呼吸训练器的应用等。甚至还有针对高危因素患者而定制的功率自行车训练等^[22, 23]^。这些均是通过术前主动干预而降低手术风险，患者会乐意且主动配合完成。这些类似的工作，只有充分的理解和沟通才能打消患者的疑虑，也才能使加速康复的效果充分显现。

ERS流程化方案使各级医生对围手术期患者的目标性评估精准和预防性措施有效，彻底改变患者认为医生就会冷冰冰的"有风险就签字"的传统观念，使治疗过程从"事后性处理"转变到"事前性预防"^[24]^。事前性预防可以使患者感受到"我被关心"而消除很多顾虑。如术前高危因素评估后，进行合理的围手术期肺康复训练及出院后常见症状的预防性管理，使患者切身感到"我的问题"都在医生的掌控中，而使医患者关系更加融洽。

外科手术实施前最常见的是术前外科医生与患者家属或/和患者进行沟通，患者是被告知的和被动选择，表现的是无奈和顾虑重重。ERAS需要多学科的协作，术前谈话不只由外科医生来完成，手术室护士及麻醉科医生都会进行术前访视，手术室护士会针对术前不置尿管，为保障术中患者安全，所采取的措施进行宣教，麻醉医生会针对患者并存疾病及可能会发生的问题，采取的保障措施与患者沟通。使患者感觉到是一个团队在为我服务。同时每个学科都会将其针对某一问题处理的最佳方案"个性化"的应用。例如：疼痛管理最能体现这种协作的实现，术前护士宣教时告知病人疼痛不需要忍受，及时告诉医生并及时处理；术中麻醉师有超前镇痛预案；外科医生会针对术后疼痛特点和部位，采取预防性处理(如切口周围的局部浸润麻醉)；术后进行基础镇痛等，最关键是术后麻醉科医生会进行镇痛效果的访视。ERAS方案使治疗过程体现"单一外科"到"多学科"与患者沟通的人文关怀。

ERAS理念也是生物医学模式(bio-medical model)转为生物-心理-社会医学模式(bio-psycho-social medical model)具体临床应用。其所体现的人文关怀体现在ERAS应用的各个方面，首先需要医生不断提高自身的技术水平并合理应用；其次ERAS方案实施需要多学科和医护康共同协作，充分体现了"以病人为中心"的理念；再次是ERAS方案临床应用效果充分体现需要患者的配合，医患的充分沟通是关键，提升就医满意度；最后是以问题为导向的团队架构，才能够充分利用现有医疗手段服务于患者，造福于患者。
